# Direct balloon dilatation angioplasty for intracranial atherosclerotic stenosis-related acute ischemic stroke: a single-arm retrospective study of technical feasibility and safety

**DOI:** 10.3389/fneur.2026.1828034

**Published:** 2026-09-16

**Authors:** Xuanfei Jiang, Xiaoyan Sun, Zhenchan Lu, Peiyin Wen, Xiaoqin Fei, Bing Zhang, Honggang Ma

**Affiliations:** 1 Department of Neurology, Huzhou Centre Hospital, Affiliated Centre Hospital Huzhou University, Huzhou City, Zhejiang, China; 2 Department of Emergency, the First Peoples Hospital of Huzhou, Huzhou City, Zhejiang, China; 3 Department of Emergency, Huzhou Centre Hospital, Affiliated Centre Hospital Huzhou University, Huzhou City, Zhejiang, China

**Keywords:** acute ischaemic stroke, angioplasty, balloon dilatation, intracranial atherosclerosis, patient safety

## Abstract

**Background:**

Direct balloon dilatation angioplasty (DBDA) is an established treatment for intracranial atherosclerotic stenosis (IAS) without acute ischemia; however, its role in IAS-induced acute ischemic stroke (ICAS-LVO) remains poorly defined. This retrospective single-arm study aimed to evaluate the technical feasibility, safety, and 90-day functional outcomes of DBDA in a selected cohort of patients with ICAS-LVO.

**Methods:**

Data from 28 consecutive patients with ICAS-LVO who underwent DBDA as the primary recanalization strategy at Huzhou Central Hospital (October 2018–December 2020) were retrospectively analyzed. Safety endpoints included intraprocedural dissection, plaque retraction, distal embolism, the need for remedial stenting, symptomatic intracranial hemorrhage (sICH), and 90-day mortality. Technical success was defined as postprocedural modified Thrombolysis in Cerebral Infarction (mTICI) grade 2b–3. Functional outcomes were assessed using the modified Rankin Scale (mRS) at 90 days.

**Results:**

Among the 28 patients (mean admission NIHSS 16.5 ± 5.6), 23 (82.1%) received intravenous thrombolysis prior to intervention. Intraprocedural dissection occurred in one patient, and plaque retraction with flow failure occurred in another patient, with both patients requiring remedial stenting. sICH was observed in one patient (3.6%); no distal embolization or 90-day mortality was observed. Successful reperfusion (mTICI 2b–3) was achieved in 26 patients (92.9%; 95% CI 77.4–98.0%), with mTICI 2b achieved in 9 (32.1%; 95% CI 17.2–51.6%) and mTICI 3 in 17 (60.7%; 95% CI 42.2–76.8%) patients. The median puncture-to-revascularization time was 65 min (IQR 55–78). The median NIHSS at discharge was 4 (IQR 2–8). At 90 days, 18 patients (64.3%; 95% CI 45.8–79.8%) achieved functional independence (mRS 0–2).

**Conclusion:**

In this highly selected cohort of ICAS-LVO patients, DBDA was associated with a high rate of successful reperfusion and favorable 90-day functional outcomes. However, given the single-arm retrospective design, the absence of a control group, and the high rate of intravenous thrombolysis use, these findings are hypothesis-generating only. The observed outcomes cannot be attributed to DBDA alone and require validation in multicenter randomized trials.

## 1 Introduction

In China, stroke has become the leading cause of death, with the incidence of acute ischemic stroke (AIS) continuously increasing (1). Intracranial atherosclerotic stenosis (IAS) is a leading cause of acute ischemic stroke (AIS), particularly when it presents as IAS-related large vessel occlusion (ICAS-LVO), in which severe stenosis leads to thrombus formation and distal hypoperfusion (2). Current guidelines recommend intravenous thrombolysis (IVT) within 4.5 h for eligible patients; however, this time window limits its application to a minority of cases, and recanalization rates for large vessel occlusions remain only 13–18% (3, 4). Although mechanical thrombectomy has revolutionized AIS treatment, its efficacy in ICAS-LVO is reduced, with failure rates reported to be as high as 77% due to re-occlusion and residual stenosis (5). Acute stenting can not only address the underlying stenosis but also introduce risks of prolonged procedure time, the requirement for dual antiplatelet therapy and associated hemorrhagic complications, and in-stent thrombosis; thus, it is generally reserved as a rescue measure (6). Consequently, there is a clinical need for a rapid and safe endovascular approach that directly targets the underlying stenotic lesion in ICAS-LVO.

Direct balloon dilatation angioplasty (DBDA) is an established technique for non-acute IAS and it offers the advantages of technical simplicity, shorter procedure time, and immediate improvement of stenosis without the mandatory use of dual antiplatelet therapy (7, 8). These characteristics make DBDA a conceptually attractive option for ICAS-LVO in the acute setting. The present study aimed to evaluate the procedural safety, technical success, and 90-day functional outcomes of DBDA as the primary recanalization strategy in a selected cohort of patients with ICAS-LVO.

## 2 Materials and methods

### 2.1 Study design and general data

This was a retrospective, single-arm, single-center observational study. The clinical data of 28 consecutive patients diagnosed with ICAS-LVO who underwent DBDA at the Stroke Center of Huzhou Central Hospital between October 2018 and December 2020 were analyzed. This study was approved by the Ethics Committee of Huzhou Central Hospital, Affiliated Central Hospital of Huzhou University. All procedures were conducted in accordance with the Declaration of Helsinki.

### 2.2 Inclusion and exclusion criteria

The diagnosis of AIS was based on the 2018 Chinese guidelines for the diagnosis and treatment of acute ischemic stroke (9).

Patients were included if they met all of the following criteria: (1) age ≥48 years; (2) acute ischemic stroke within 6 h of symptom onset with a National Institutes of Health Stroke Scale (NIHSS) score of ≥6; (3) pre-stroke modified Rankin Scale (mRS) score of 0–1; (4) CT angiography (CTA) and digital subtraction angiography (DSA) demonstrating severe intracranial atherosclerotic stenosis (≥70% according to the WASID criteria) involving the intracranial internal carotid artery (ICA), M1 segment of the middle cerebral artery (MCA), intracranial vertebral artery (VA), or basilar artery (BA), causing large vessel occlusion or critical hypoperfusion, without tandem extracranial stenosis of ≥50%; (5) arterial puncture performed within 6 h of symptom onset; and (6) signed informed consent for the interventional procedure obtained from the patient’s legal representative.

The exclusion criteria were as follows: (1) tandem intracranial and extracranial lesions (extracranial stenosis ≥50%); (2) known contrast allergy; (3) severe arterial tortuosity precluding stable balloon positioning; (4) coexistent intracranial aneurysms or arteriovenous malformations requiring treatment; (5) active malignancy, autoimmune disease, or a known bleeding disorder; (6) significant organ dysfunction (e.g., renal or hepatic failure); (7) life expectancy of <6 months, as determined by the treating physician based on the patient’s pre-stroke comorbidities and clinical status; (8) platelet count of <50 × 10^9^/L.

#### 2.2.1 Baseline imaging assessment

Pre-procedural non-contrast CT was used to assess the Alberta Stroke Program Early CT Score (ASPECTS) in patients with anterior circulation occlusions by a neuroradiologist blinded to clinical outcomes. Collateral circulation was graded on CT angiography according to the American Society of Interventional and Therapeutic Neuroradiology/Society of Interventional Radiology (ASITN/SIR) scale (grades 0–4), with grades 3–4 considered indicative of good collateral status.

### 2.3 Intervention procedure

DBDA was performed as the primary recanalization strategy; no stent-retriever, aspiration thrombectomy, or any other mechanical thrombectomy device was used during the procedure. All patients underwent the procedure under general anesthesia. For patients who had received IVT, systemic heparinization was withheld; in those without prior IVT, intravenous heparin (50–70 IU/kg bolus) was administered to achieve an activated clotting time of 250–300 s. A semi-compliant intracranial balloon [including Gateway (Medtronic) and Balloonfish] was selected with a diameter of 50–70% of the normal target vessel diameter and a length sufficient to cover both ends of the lesion (mean lesion length 8.5 ± 3.2 mm). Submaximal angioplasty was performed with gradual inflation at 1 atm per minute to 80–90% of the nominal pressure, maintained for 30–60 s, for 1–2 inflations. Intraoperative blood pressure was maintained between 120–140/70–90 mm Hg. The balloon was then deflated slowly (1 atm/15 s), and a control angiogram was obtained through the retained microguidewire to assess antegrade flow, residual stenosis, and evidence of dissection or plaque retraction. If antegrade flow was unstable or vessel re-occlusion occurred within 30 min, rescue stenting was performed.

A non-contrast cranial CT was performed 24-h post-procedure to detect intracranial hemorrhage. In patients without hemorrhage, single antiplatelet therapy (aspirin 100 mg or clopidogrel 75 mg daily) was initiated 24 h after the procedure. For those who underwent stent placement, dual antiplatelet therapy (aspirin 100 mg daily plus clopidogrel 300 mg loading dose by 75 mg daily) was administered.

### 2.4 Observation indicators and outcome measures

Safety endpoints were defined as follows: (1) intraprocedural vessel dissection, (2) plaque retraction with flow failure, (3) angiographic distal embolism, (4) requirement for rescue stenting, (5) symptomatic intracranial hemorrhage (sICH), defined as any intracranial hemorrhage on 24-h non-contrast CT with an increase in NIHSS score of ≥4 points, according to the ECASS III criteria (10), and assessed by an independent radiologist, and (6) 90-day all-cause mortality.

The primary technical endpoint was successful reperfusion, defined as a postprocedural modified Thrombolysis in Cerebral Infarction (mTICI) grade of 2b or 3 (11). The primary clinical endpoint was functional independence at 90 days, defined as a modified Rankin Scale (mRS) score of 0–2.

Secondary endpoints comprised: (1) time from femoral artery puncture to revascularization, (2) change in NIHSS score from admission to discharge, and (3) procedure-related sICH rate.

### 2.5 Statistical methods

Data were analyzed using SPSS (version 20.0.IBM Corp, Armonk, NY, USA) Sprinter balloon catheter, Medtronic, Minneapolis, MN, USA Enterprise vascular reconstruction device and delivery system, Codman & Shurtleff, Raynham, MA, USA. Continuous variables are presented as mean ± standard deviation or median (interquartile range) as appropriate. Categorical variables are expressed as frequencies and percentages with 95% confidence intervals (CI) calculated using the Wilson method. Because NIHSS scores are ordinal and typically non-normally distributed, the change from admission to discharge was assessed using the Wilcoxon signed-rank test, with a median difference, Hodges–Lehmann estimator, and associated 95% CI reported. A two-sided *p*-value of < 0.05 was considered statistically significant.

## 3 Results

### 3.1 Baseline characteristics

A patient selection flowchart is shown in Figure 1.

**Figure 1:**
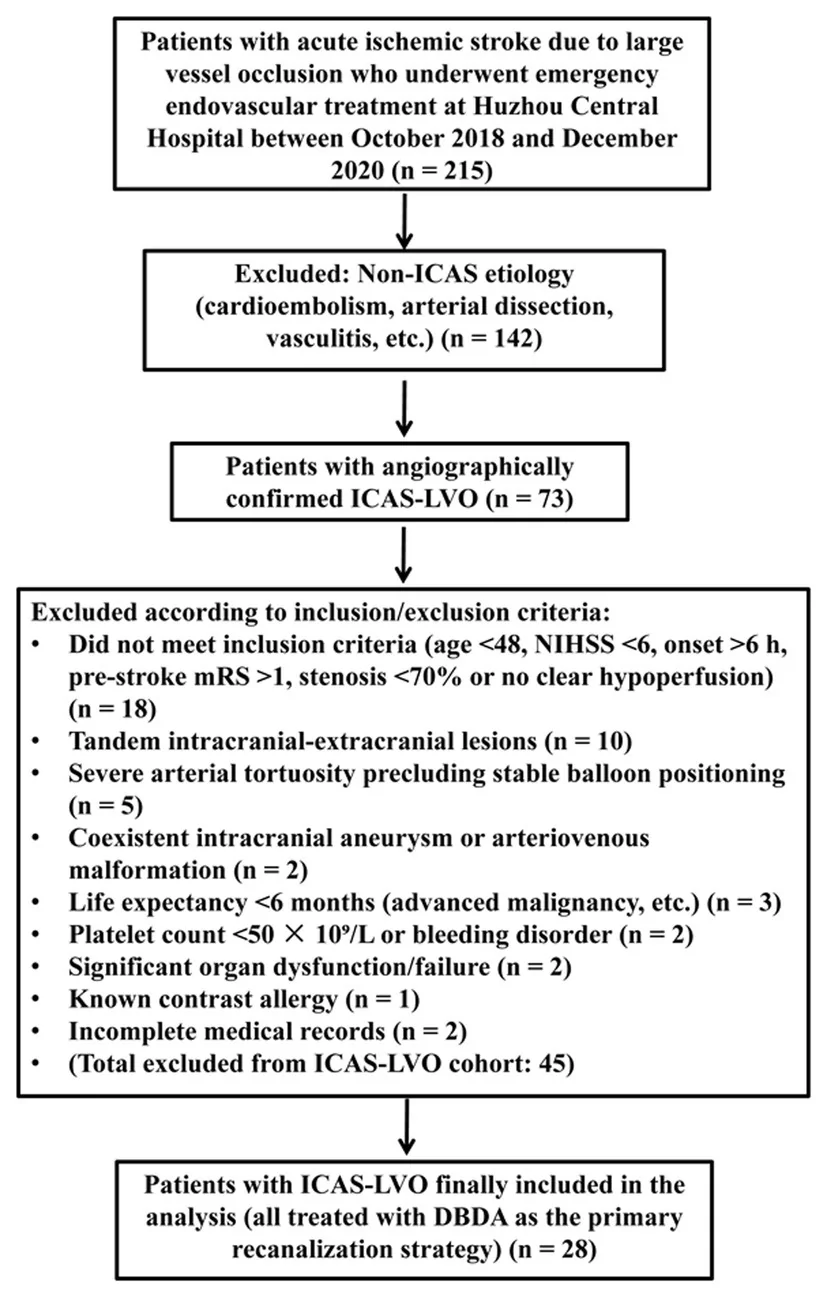
Patient selection flowchart. ICAS-LVO, intracranial atherosclerotic stenosis-related large vessel occlusion; DBDA, direct balloon dilatation angioplasty; NIHSS, National Institutes of Health Stroke Scale; mRS, modified Rankin Scale.

The baseline demographic and clinical characteristics of the 28 patients are summarized in Table 1. The median age was 63 years (IQR 55–71), and 18 (64.3%) were male. Hypertension was present in 21 (75.0%), diabetes in 9 (32.1%), atrial fibrillation in 4 (14.3%), and 11 (39.3%) were smokers. The mean NIHSS score on admission was 16.54 ± 5.57 (median 17, IQR 12–20), and all patients had a pre-stroke mRS score of 0. The mean time from symptom onset to arterial puncture was 145.2 ± 25.6 min and from onset to revascularization was 212.6 ± 32.1 min. Intravenous alteplase was administered to 23 of 28 patients (82.1%) before the intervention. The occlusion sites in the overall cohort (*n* = 28) were as follows: intracranial ICA in 5 (17.9%), M1 MCA in 14 (50.0%), V4 VA in 2 (7.1%), and basilar artery in 7 (25.0%). The mean hospitalization duration was 12.25 ± 5.49 days. Baseline systolic blood pressure (mean ± SD) was 148 ± 22 mm Hg; no significant correlation between admission blood pressure and NIHSS was observed (Spearman’s ρ = 0.12, *p* = 0.54). Among the 19 patients with anterior circulation occlusion, the median baseline ASPECTS was 9 (IQR 8–10). For the 9 patients with posterior circulation occlusion, pre-procedural non-contrast CT was used to qualitatively assess the extent of early ischemic changes; no formal posterior circulation ASPECTS (pc-ASPECTS) was calculated. Good collateral status (ASITN/SIR grade 3–4) was present in 18 patients (64.3%), while 8 patients (28.6%) had intermediate collaterals (grade 2), and 2 patients (7.1%) had poor collaterals (grade 1).

**Table 1:** Baseline characteristics of the 28 patients [*n* (%)].

Variables	DBDA (*n* = 28)
Age (years)	62.9 ± 10.7
Male sex	18 (64.3%)
Female sex	10 (35.7%)
Hypertension	21 (75.0%)
Diabetes mellitus	9 (32.1%)
Atrial fibrillation	4 (14.3%)
Current smoking	11 (39.3%)
Admission NIHSS score, mean ± SD	16.5 ± 5.6
Admission NIHSS score, median (IQR)	17 (12–20)
Pre-stroke mRS 0	28 (100%)
Admission systolic blood pressure (mm Hg), mean ± SD	148 ± 22
ASPECTS (anterior circulation only, *n* = 19), median (IQR)	9 (8–10)
Collateral grade (ASITN/SIR 0–4), *n* (%)	
0	0 (0%)
1	2 (7.1%)
2	8 (28.6%)
3	12 (42.9%)
4	6 (21.4%)
Good collaterals (grade 3–4)	18 (64.3%)
Time from symptom onset to femoral artery puncture (min), mean ± SD	145.2 ± 25.6
Time from admission to femoral artery puncture (min), mean ± SD	99.1 ± 12.5
Time from admission to revascularization (min), mean ± SD	166.1 ± 21.4
Intravenous alteplase (rt-PA) administered	23 (82.1%)
Occlusion site	
Intracranial ICA	5 (17.9%)
MCA M1 segment	14 (50.0%)
VA V4 segment	2 (7.1%)
Basilar artery	7 (25.0%)
Length of hospital stay (days), mean ± SD	12.3 ± 5.5

NIHSS, National Institutes of Health Stroke Scale; mRS, modified Rankin Scale; ASPECTS, Alberta Stroke Program Early CT Score; ASITN/SIR, American Society of Interventional and Therapeutic Neuroradiology/Society of Interventional Radiology; ICA, internal carotid artery; MCA, middle cerebral artery; VA, vertebral artery.

### 3.2 Safety evaluation

Periprocedural adverse events are listed in Table 2. Of the 28 procedures, intraprocedural vessel dissection occurred in 1 patient (Case 4), and plaque retraction with compromised antegrade flow occurred in 1 patient (Case 3), both necessitating rescue stenting. Thus, 2 of 28 patients (7.1%) received a stent as a rescue measure. sICH occurred in one patient (3.6%); no angiographic distal embolization or 90-day mortality was observed. The overall rate of periprocedural adverse events was 10.7% (3/28; 95% CI 3.7–26.1%).

**Table 2:** Intraprocedural adverse events (*n* = 28).

Variables	*n* (%)
Vascular dissection requiring remedial stenting	1 (3.6%)
Plaque retraction with flow failure requiring remedial stenting	1 (3.6%)
Remedial stenting (total)	2 (7.1%)
Symptomatic intracranial hemorrhage	1 (3.6%)
90-day mortality	0 (0%)
Any intraprocedural adverse event	3 (10.7%)

In addition to these adverse events, 2 of the 28 patients (7.1%) had unsuccessful reperfusion (final mTICI grade <2b). In one patient, the balloon could not be advanced across a severely calcified lesion in the distal vertebral artery despite multiple attempts; the procedure was terminated without further intervention, and the patient was managed with best medical therapy. In the other patient, balloon dilatation was performed but failed to achieve meaningful antegrade flow improvement (mTICI 1); the operator decided against rescue stenting due to concern for hemorrhagic transformation given a large baseline infarct core. Both cases were included in the primary analysis (*N* = 28) and were classified as technical failures. Neither of these two cases met the criteria for procedural adverse events—dissection, plaque retraction, or sICH, as defined in Section 2.4, and they were therefore not listed in Table 2.

### 3.3 Reperfusion and functional outcomes

The procedure was technically successful (final mTICI 2b–3) in 26 of 28 patients, yielding a success rate of 92.9% (95% CI 77.4–98.0%). Postprocedural mTICI 2b was achieved in 9 patients (32.1%; 95% CI 17.2–51.6%), and mTICI 3 in 17 patients (60.7%; 95% CI 42.2–76.8%). The mean puncture-to-revascularization time was 67.50 ± 18.29 min (median 65, IQR 55–78).

At discharge, the median NIHSS score was 4 (IQR 2–8), representing a significant reduction from admission (median difference −11, 95% CI − 14 to −8; Wilcoxon signed-rank test; Z = −4.58, *p* < 0.001).

The 90-day mRS distribution was as follows: 0 in 6 (21.4%), 1 in 9 (32.1%), 2 in 3 (10.7%), 3 in 2 (7.1%), 4 in 7 (25.0%), and 5 in 1 (3.6%). Overall, 18 of 28 patients (64.3%; 95% CI 45.8–79.8%) achieved functional independence (mRS 0–2) at 90 days.

### 3.4 Representative cases

A summary of four representative cases is provided below. Successful cases typically had lesion lengths of <10 mm and favorable collateral status, whereas rescue stenting was indicated for dissection or plaque retraction, with immediate DSA confirmation and flow restoration (Figures 2–5).

**Figure 2:**
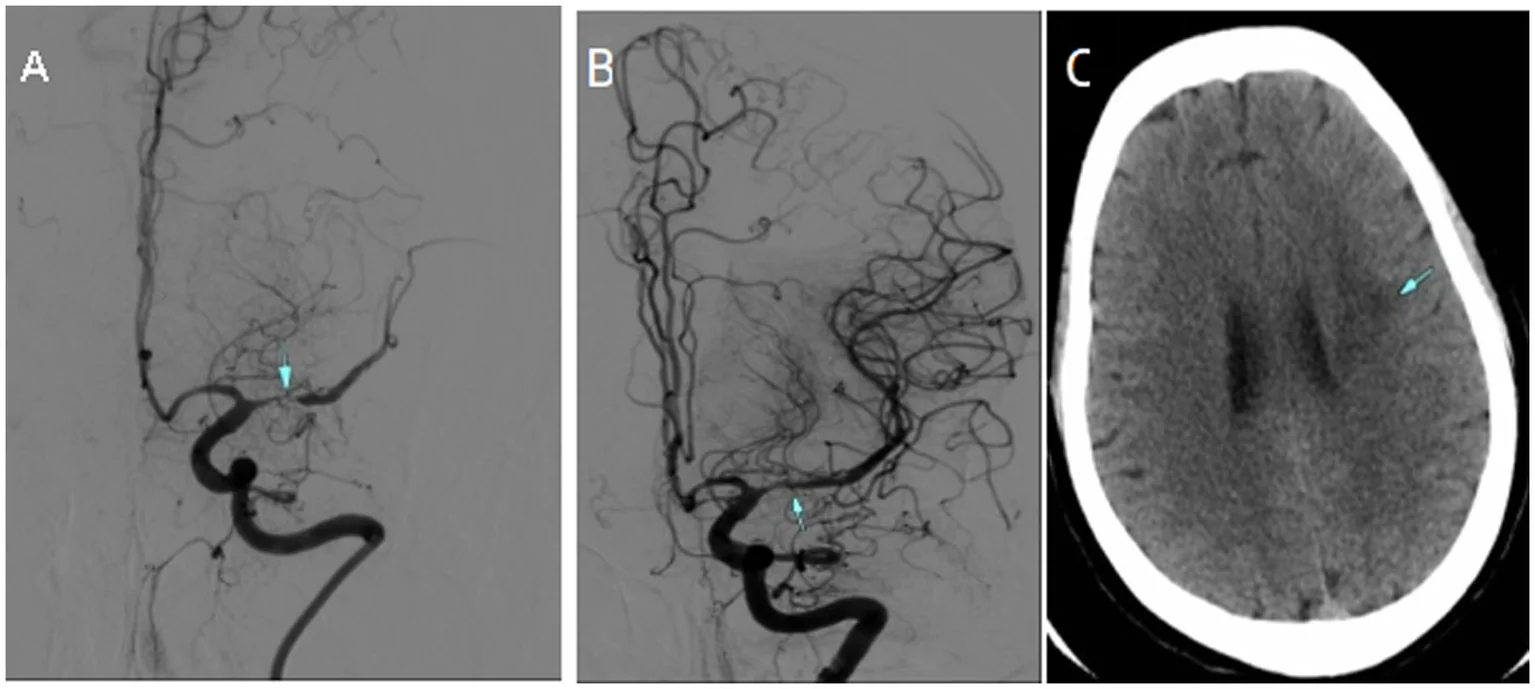
Clinical data of case 3. **(A)** DSA shows severe stenosis at the M1 segment of the left middle cerebral artery (arrow) with distal flow restriction. **(B)** Improved stenosis after balloon dilatation (arrow). **(C)** Cranial CT 24-h post-procedure demonstrates a small infarct in the left frontoparietal lobe (arrow).

**Figure 3:**
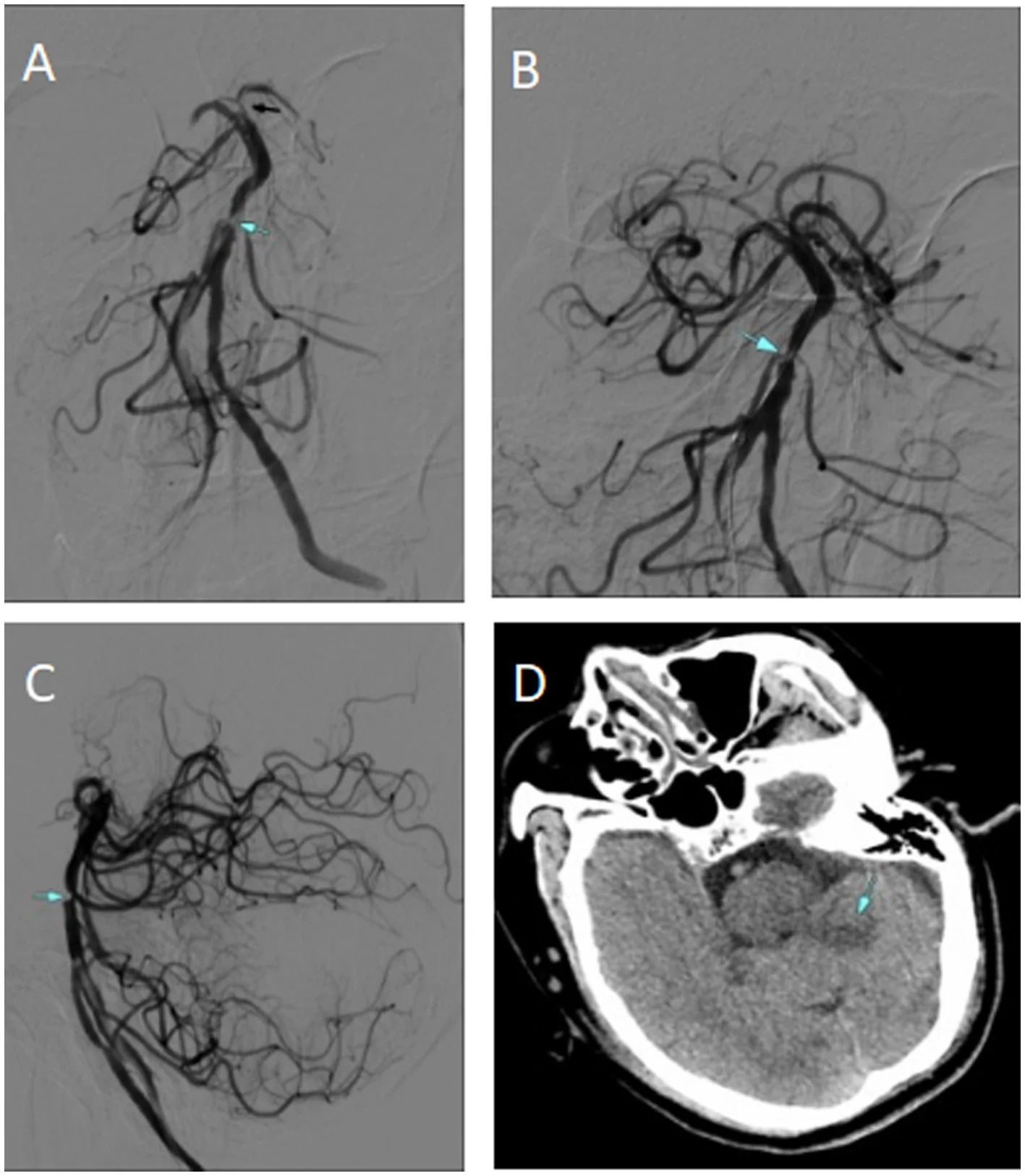
Clinical data of case 2. **(A)** DSA orthogonal view reveals severe stenosis of the middle and lower basilar arteries (green arrow) and a thrombus in the distal basilar artery (black arrow). **(B)** After balloon dilatation, the previously visible thrombus is no longer seen, and distal blood flow has improved. **(C)** Antegrade flow remains unaffected despite residual stenosis (arrow). **(D)** Cranial CT 24 h post-procedure shows a small infarct in the left cerebellar hemisphere (arrow).

**Figure 4:**
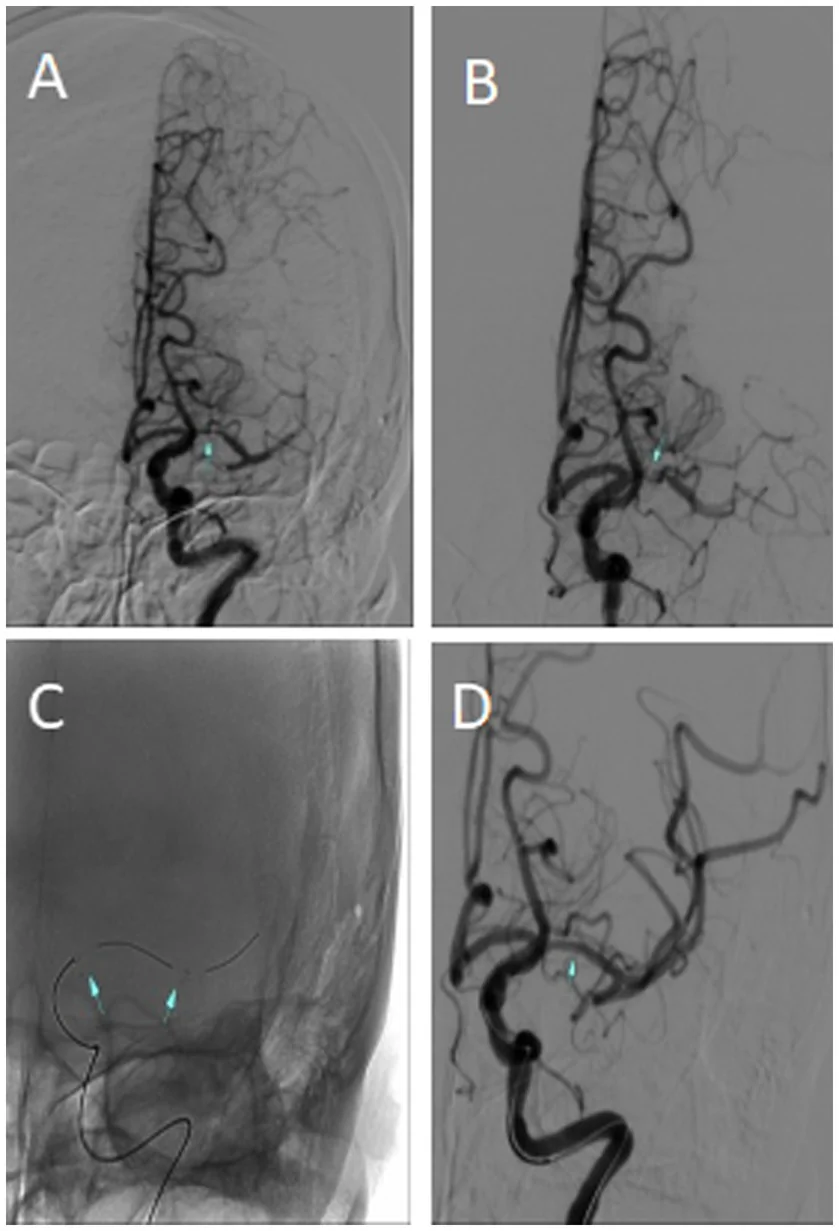
Clinical data of case 3. **(A)** DSA demonstrates severe stenosis from the distal internal carotid artery to the origin of the middle cerebral artery (arrow). **(B)** Rescue stenting procedure. **(C)** Post-dilation angiography reveals plaque retraction with compromised antegrade flow (arrow). **(D)** Improved stenosis after rescue stenting (arrow).

**Figure 5:**
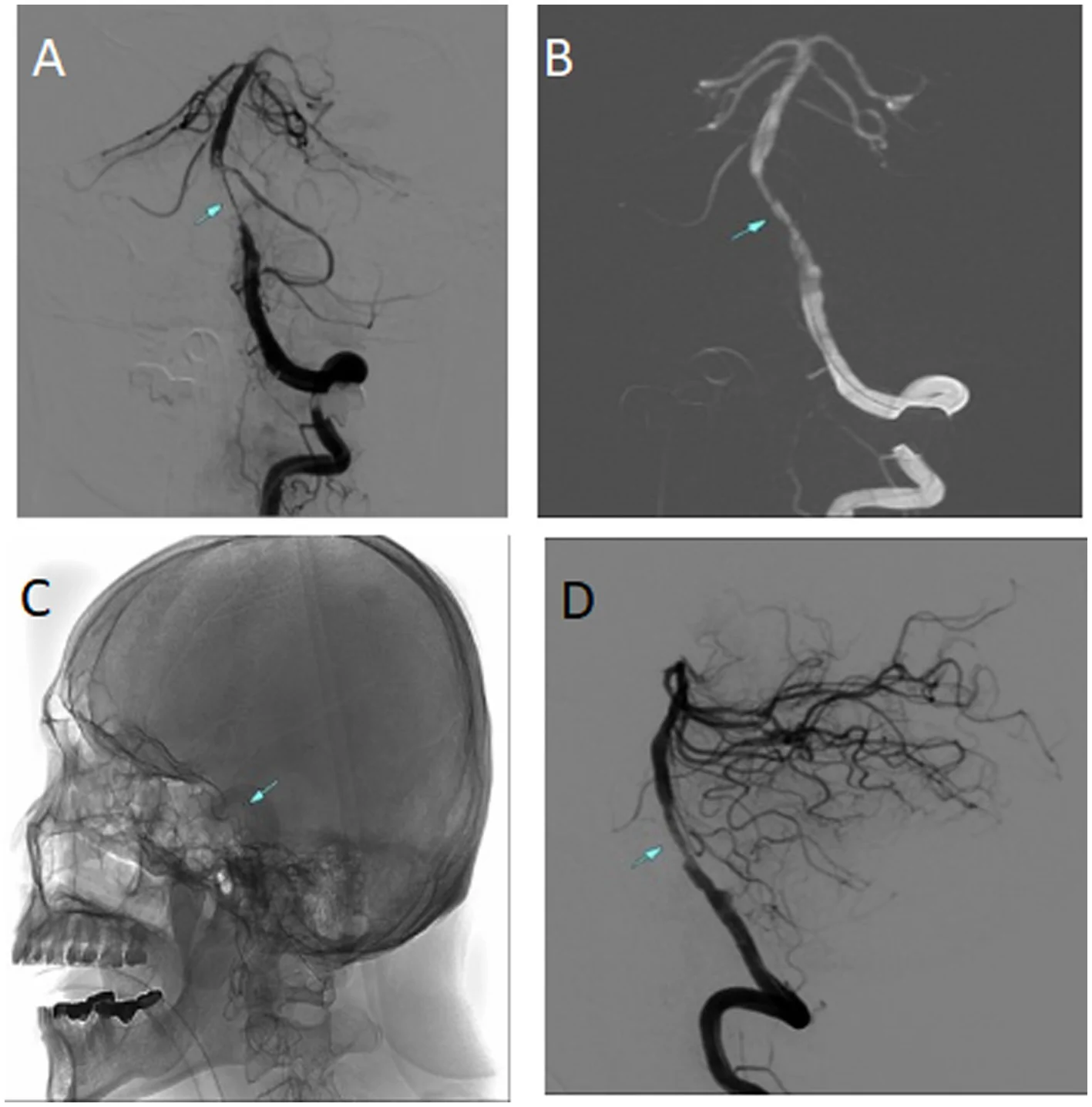
Clinical data of case 4. **(A)** DSA shows severe stenosis at the V4 segment of the left vertebral artery and the inferior basilar artery **(B)** Post-dilation angiography reveals vascular dissection with flow limitations (arrow) **(C)** Rescue stenting procedure (arrow) **(D)** Significant improvement in stenosis and restoration of distal blood flow after rescue stenting (arrow).

Case 1: A patient in their mid-60s was admitted to our hospital with a complaint of “sudden onset of right-side limb weakness.” The NIHSS score was 10 on admission. DSA showed severe stenosis in the M1 segment of the left middle cerebral artery with distal flow restriction (Figure 2A). The patient underwent a DBDA (with a 2.0 mm × 15 mm balloon) procedure. Post-dilation angiography demonstrated improved stenosis (Figure 2B). Cranial CT at 24 h indicated a small infarct in the left frontoparietal lobe (Figure 2C). There was a substantial improvement in antegrade flow, with a final mTICI grade of 3. The NIHSS score at discharge was 1, and the 90-day mRS score was 1.

Case 2: A patient in their early 60s was admitted to our hospital with a complaint of “sudden onset of unconsciousness for 34 min.” The NIHSS score was 25 on admission. DSA orthogonal views showed severe stenosis of the middle and lower basilar arteries (Figure 3A). A thrombus was visible at the end of the basilar artery. The patient underwent a DBDA (with a 2.0 mm × 15 mm balloon) procedure. After dilatation, the distal blood flow improved significantly, and the previously noted thrombus was no longer visible, which may be attributable to the mechanical compression of the thrombus against the atherosclerotic plaque by the balloon, with distal washout of fragmented microthrombi into the microcirculation, which may be augmented by intravenous thrombolysis. No angiographically evident distal embolization was observed (Figure 3B). Despite residual stenosis, antegrade flow remained unaffected (Figure 3C). Cranial CT at 24 h showed a small infarct in the left cerebellar hemisphere (Figure 3D). The NIHSS score at discharge was 1, and the 90-day mRS score was 0.

Case 3: A patient in their late 40s was admitted to our hospital with a complaint of sudden inability to speak and right limb immobility for more than 3 h. The NIHSS score was 22 on admission. DSA showed severe stenosis from the end of the internal carotid artery to the starting point of the middle cerebral artery (Figure 4A). The patient underwent a DBDA (with a 2.0 mm × 15 mm balloon) procedure. Post-dilation angiography revealed plaque retraction with compromised antegrade flow and a failure to sustain vessel patency (Figure 4C); therefore, rescue stenting was performed (Figures 4B,D). Stent deployment improved the stenosis and restored distal perfusion. The final mTICI grade was 3. At discharge, the NIHSS score was 3, and the 90-day mRS score was 0.

Case 4: A patient in their early 50s was admitted to our hospital with a complaint of sudden loss of consciousness for 4 h. The NIHSS score was 26 on admission. DSA suggested severe stenosis in the V4 segment of the patient’s left vertebral artery and the inferior basilar artery (Figure 5A). The patient underwent a DBDA (with a 2.0 mm × 15 mm balloon) procedure. Post-dilation angiography showed vascular dissection with flow limitations (Figure 5B). Consequently, rescue stenting was performed (Figure 5C), which led to a significant improvement in the stenosis and restoration of distal blood flow (Figure 5D). The final mTICI grade was 3. At discharge, the NIHSS score was 12, and the 90-day mRS score was 4.

## 4 Discussion

This single-arm, retrospective study suggests that, in a highly selected cohort of patients with ICAS-LVO, DBDA as the primary recanalization strategy is technically feasible, associated with a high rate of successful reperfusion (92.9%), and may yield favorable 90-day functional outcomes (mRS 0–2 in 64.3%). However, these findings should be interpreted with extreme caution. The absence of a control group, the large proportion of patients receiving intravenous thrombolysis (82.1%), and the deliberate selection of patients with favorable baseline imaging characteristics (small core, incomplete occlusion, and good collaterals) prevent any attribution of improved outcomes to DBDA itself. As such, the present results are best viewed as hypothesis-generating, underscoring the need for prospective randomized studies.

The safety profile of DBDA in this study appears acceptable, with a 7.1% rate of rescue stenting for dissection/retraction and 3.6% incidence of sICH, and no 90-day mortality. These complication rates are comparable to or lower than those reported for acute stenting in ICAS-LVO (12, 13). The use of submaximal angioplasty with undersized balloons likely contributed to the low incidence of vessel wall injury. Nevertheless, the observed 0% mortality in a cohort with a median admission NIHSS of 17—including basilar and terminal ICA occlusions—is implausibly favorable for an unselected ICAS-LVO population and almost certainly reflects the strict inclusion and exclusion criteria, which selected patients with small core infarcts, good leptomeningeal collaterals, and the absence of tandem lesions. This profound selection bias severely limits the generalizability of our findings and represents the single most important limitation of this study.

Previous studies on direct angioplasty for ICAS-LVO have reported similar 90-day functional independence rates. Zhang et al. (14) performed DBDA in patients with a mean baseline NIHSS of 14.9 and found that 60.3% achieved mRS 0–2 at 90 days. Luo et al. (15) reported a 56.1% rate of 90-day functional independence after direct angioplasty for acute middle cerebral artery occlusion with good collateral status. In the present series, the rate of 90-day mRS 0–2 was 64.3%, which is likely inflated by our deliberate inclusion of patients with incomplete occlusion and small baseline cores. Notably, 82.1% of our patients received intravenous alteplase, which independently improves outcomes and represents a major confounder that cannot be statistically disentangled in a single-arm design. Any neurological improvement observed cannot be distinguished between the effects of DBDA, thrombolysis, and natural penumbral recovery. Furthermore, the shorter procedure time associated with DBDA (median 65 min) compared with historical stenting times (~155 min) (12) may be advantageous; however, this finding requires confirmation in comparative studies.

This study has several critical limitations beyond its retrospective, single-arm design and small sample size. First, the strict selection criteria—including the exclusion of tandem lesions, severe tortuosity, and patients with large completed infarcts—strongly bias the results toward favorable outcomes, as discussed above. Second, the absence of systematic CT perfusion data precludes any conclusions regarding penumbral salvage. Third, 82.1% of patients received IVT, constituting a major confounder. Fourth, the lack of a control group (either medical management or thrombectomy alone) makes it impossible to isolate the effects of DBDA. Fifth, long-term outcomes beyond 90 days, including restenosis rates, were not assessed. Therefore, the present findings should only be regarded as preliminary evidence to support the design of future multicenter randomized controlled trials.

## 5 Conclusion

In this selected series of 28 patients with ICAS-LVO, DBDA was technically feasible and was associated with a high reperfusion rate and favorable 90-day functional outcomes. However, the study’s single-arm retrospective design, substantial selection bias, and the predominant use of intravenous thrombolysis preclude any conclusions regarding efficacy. These hypothesis-generating observations warrant further investigation within the context of multicenter, prospective, randomized controlled trials.

## Data Availability

The original contributions presented in the study are included in the article/supplementary material, further inquiries can be directed to the corresponding author.
